# Supplementary material for: Mortality predictors, hepatic involvement patterns, and the steatotic liver paradox in 1,484 hospitalized Dengue patients
Source: PLoS One. 2026 Apr 30;21(4):e0348232. doi: 10.1371/journal.pone.0348232 (PMC13132437; doi:10.1371/journal.pone.0348232)
Supplement: S1 Text — Word document representing all the missing data for all key analytical variables of patients included in the study. (DOCX) [file pone.0348232.s001.docx]

**S1 Table:** Missing Variables Dataset.

Reporting the missing data for all key analytical variables of patients included in the study.

| **Variable** | **Available** | **Missing** | **% Missing** |
| --- | --- | --- | --- |
| Age, Sex, Hb, WBC, Platelets | 1,484 | 0 | 0.0% |
| Platelets (nadir) | 1,484 | 0 | 0.0% |
| AST | 1,472 | 12 | 0.8% |
| ALT | 1,473 | 11 | 0.7% |
| NLR | 1,481 | 3 | 0.2% |
| CRP | 1,452 | 32 | 2.2% |
| Total bilirubin | 1,449 | 35 | 2.4% |
| Creatinine | 1,438 | 46 | 3.1% |
| Albumin | 1,419 | 65 | 4.4% |
| BMI | 1,169 | 315 | 21.2% |
| Ferritin | 1,140 | 344 | 23.2% |
| INR | 599 | 885 | 59.6% |
| SOFA/SAPS-3* | 188 | 1,296 | 87.3% |

**SOFA and SAPS-3 scores were calculated only for ICU patients (n=188, 12.7%), consistent with their intended clinical application. These are not “missing” in the conventional sense.*
