# Supplementary material for: Mortality predictors, hepatic involvement patterns, and the steatotic liver paradox in 1,484 hospitalized Dengue patients
Source: PLoS One. 2026 Apr 30;21(4):e0348232. doi: 10.1371/journal.pone.0348232 (PMC13132437; doi:10.1371/journal.pone.0348232)
Supplement: Attachment — Submitted filename: Peer Review Report.docx [file pone.0348232.s002.docx]

**Peer Review Report (PONE-D-26-03708 )**

**Manuscript Title:**
Clinical Outcomes, Machine Learning-Derived Phenotypes, Mortality Predictors, Hepatic Involvement Patterns and the Steatotic Liver Paradox in 1,484 Hospitalized Patients with Dengue.

Overall, this is a well-designed study involving a cohort of 1,484 patients. The statistical analysis is robust, and the discussion is well-structured and comprehensive. The conclusions are appropriate and evidence based. The references are extensive and up to date. However, the article would benefit from some revisions.

**Major Comments**

1. **Definition of Liver Involvement Groups**

Patients were categorized into:

1. Pre-existing chronic liver disease
2. Non-CLD liver involvement (steatotic liver disease)
3. No liver involvement

However, the diagnostic criteria for steatotic liver disease is not clearly defined. It is unclear whether MASLD was diagnosed based on:

- Imaging
- Clinical history
- Laboratory markers
- Metabolic syndrome criteria

Given that MASLD diagnosis typically requires imaging or histologic evidence, clearer criteria are essential to avoid misclassification bias.

2. **Conceptual Interpretation of the “Steatotic Liver Paradox” or “Obesity paradox”**

The authors report that patients with steatotic liver disease had better survival than those without liver disease, suggesting an “obesity paradox.” However, this conclusion may be premature due to several possible confounders:

- Younger age distribution in MASLD group
- Admission threshold bias
- Differences in comorbidity burden
- Selection bias inherent in single-center studies

The manuscript should perform propensity-adjusted analysis specifically assessing MASLD vs non-MASLD before asserting a protective effect.

**Minor Comments**

**1. Title:** Title is too long and includes redundant elements( listing every main finding)

**2 . Terminology Consistency**

The manuscript uses multiple terms interchangeably:

- Steatotic liver disease
- MASLD
- Metabolic syndrome

Clear definitions and consistent terminology are needed.

**3. Acute Kidney Injury Definition**

AKI is defined as creatinine >1.5 mg/dL at admission, which deviates from KDIGO criteria. The authors should justify this simplified definition .

**4. Reporting of Missing Data**

The authors state that complete case analysis was performed but should provide:

- Percentage of missing data for each variable
- Sensitivity analyses results

4. **Figure and Table Density**

The manuscript includes many figures that repeat information presented in tables. Condensing some figures into supplementary materials would improve readability.

**Suggestions for Improvement**

1. Make the title concise and remove redundant elements.
2. Provide clear diagnostic criteria for steatotic liver disease (MASLD).
3. Adjust the analysis evaluating the steatotic liver paradox or obesity paradox using propensity matching or multivariable models.
4. Adopt standard AKI definitions (KDIGO).
5. Provide detailed missing data reporting.

**Overall Recommendation**

**Minor Revision**

The manuscript addresses an important topic and contains a valuable dataset. However, several methodological and analytical issues must be clarified or corrected before the findings can be considered robust and generalizable.
