# Supplementary material for: Mortality predictors, hepatic involvement patterns, and the steatotic liver paradox in 1,484 hospitalized Dengue patients
Source: PLoS One. 2026 Apr 30;21(4):e0348232. doi: 10.1371/journal.pone.0348232 (PMC13132437; doi:10.1371/journal.pone.0348232)
Supplement: Attachment — Submitted filename: PEER REVIEW REPORT. PONE-D-26-03708R1.docx [file pone.0348232.s004.docx]

**PEER REVIEW REPORT (PONE-D-26-03708R1)**

The authors have comprehensively addressed all previously raised concerns. The manuscript has improved significantly in methodological clarity, analytical rigor, and overall scientific quality.

1. The definition of liver involvement groups is now clearly described with ultrasonographic criteria.
2. Steatotic Liver Paradox Analysis: Robustly addressed with appropriate propensity-based analyses and expanded multivariable modeling, strengthening the validity of conclusions.
3. Minor issues related to presentation, terminology, AKI definition, and missing data reporting have also been satisfactorily resolved.

Overall, I have no further concerns.

**Recommendation: Accept**
